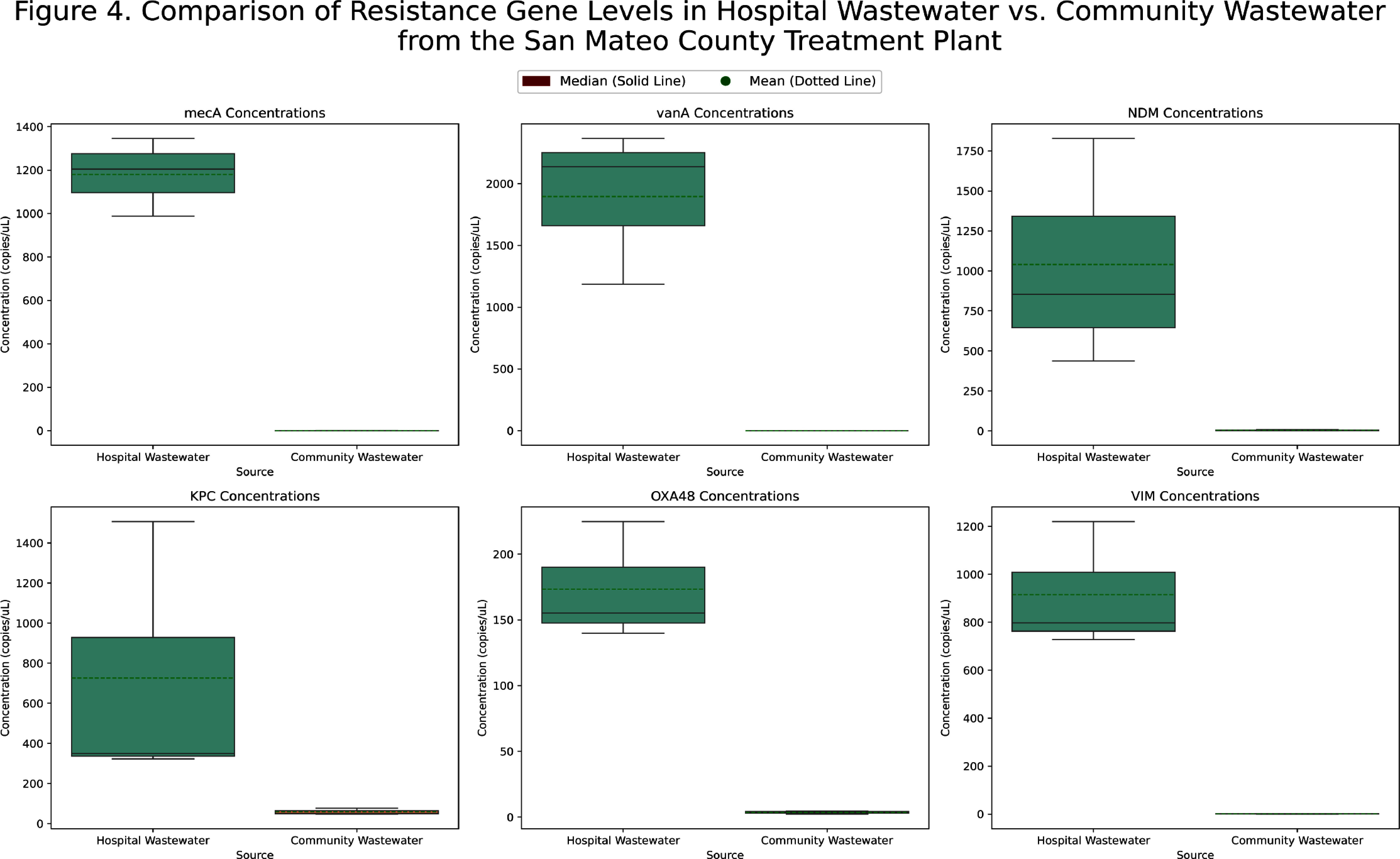# Lessons from Implementing Wastewater-Based Epidemiological Monitoring in a Northern California Acute Care Hospital, June–July 2024

**DOI:** 10.1017/ash.2025.229

**Published:** 2025-09-24

**Authors:** Guillermo Rodriguez Nava, Alessandro Zulli, Matt Grieshop, Erika Paola Viana Cardenas, Sehee Jong, Eugenia Miranti, Mingjun Jiang, Alvaro Ayala, Mindy Sampson, Ami Bhatt, Alexandria Boehm, Jorge Salinas

**Affiliations:** 1 Stanford University School of Medicine; 2 Stanford University; 3 Stanford Health Care

## Abstract

**Background:** Wastewater-based epidemiology has demonstrated effectiveness in monitoring trends of viral infections at the city, state, and national levels. It captures data independent of testing intensity, providing a comprehensive biological sample of pathogens excreted in all secretions, that is unaffected by individual testing behaviors. Traditional healthcare-associated infection surveillance relies on case-based approaches, which can be resource-intensive, prone to misclassification, and may miss patients who are colonized. We aimed to evaluate the feasibility of implementing wastewater-based epidemiology in an acute care hospital for monitoring pathogens relevant to infection prevention and control. **Methods:** In this pilot study, we deployed a Teledyne ISCO 5800™ wastewater autosampler to collect weekly composite 1000 mL samples (15 mL every 151 minutes) from the final Stanford Hospital outflow point before wastewater merged with the community system. Wastewater samples were processed within 48 hours of collection. The solid phase was separated via centrifugation, followed by nucleic acid extraction employing silica-based purification techniques optimized for efficient inhibitor removal. Droplet digital PCR was conducted targeting pathogens previously validated by the WastewaterSCAN program (https://www.wastewaterscan.org/en/pathogens). We compared hospital wastewater nucleic acid concentrations with the number of positive tests/cultures at Stanford Hospital during the same period and with Wastewaterscan community wastewater data. **Results:** We collected three weekly composite samples: Jun 20–26, Jul 10–17, and Jul 18–25. Challenges included the location of the final outflow, and the autosampler’s size (132 x 74 x 84 cm and 88.5 kg). The outflow point was situated in a high-traffic area for patients and staff, requiring barricades to ensure safety and prevent interference with sampling equipment. In terms of interpreting results, viral nucleic acid concentrations (e.g., influenza, SARS-CoV-2) appeared to parallel the number of clinical cases and were similar to community wastewater trends (Figure 1). Most antimicrobial resistance genes, including vanA (Figure 2) and carbapenemase genes (KPC, NDM, OXA-48, VIM) (Figure 3), showed limited alignment with clinical cases; however, mecA exhibited some alignment (Figure 2). Hospital wastewater had higher resistance gene concentrations than community wastewater from San Mateo County (Figure 4). **Conclusion:** Continuous collection of hospital wastewater proved challenging, mainly from logistical issues such as equipment size and access limitations. Clinical respiratory virus trends appeared to be reflected in wastewater data. However, trends for antimicrobial resistance genes may be influenced by additional factors, such as the number of colonized patients, bacterial load in the hospital sewage system, hospital antimicrobial use, and antibiotic residues in wastewater.

**Figure:**
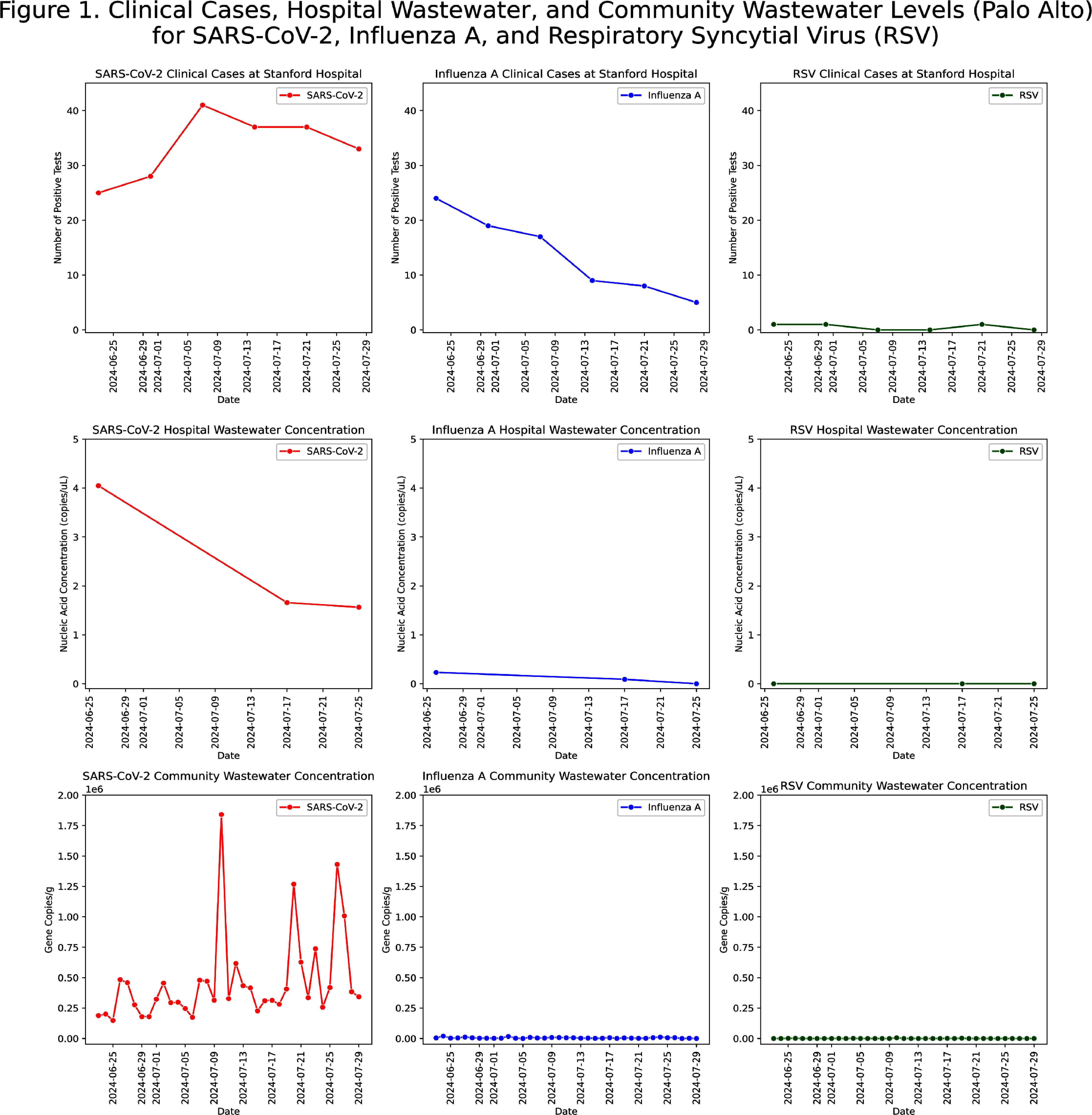

**Figure:**
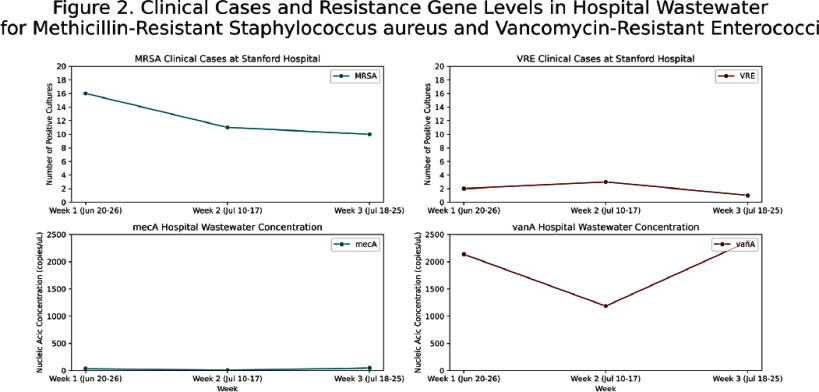

**Figure:**
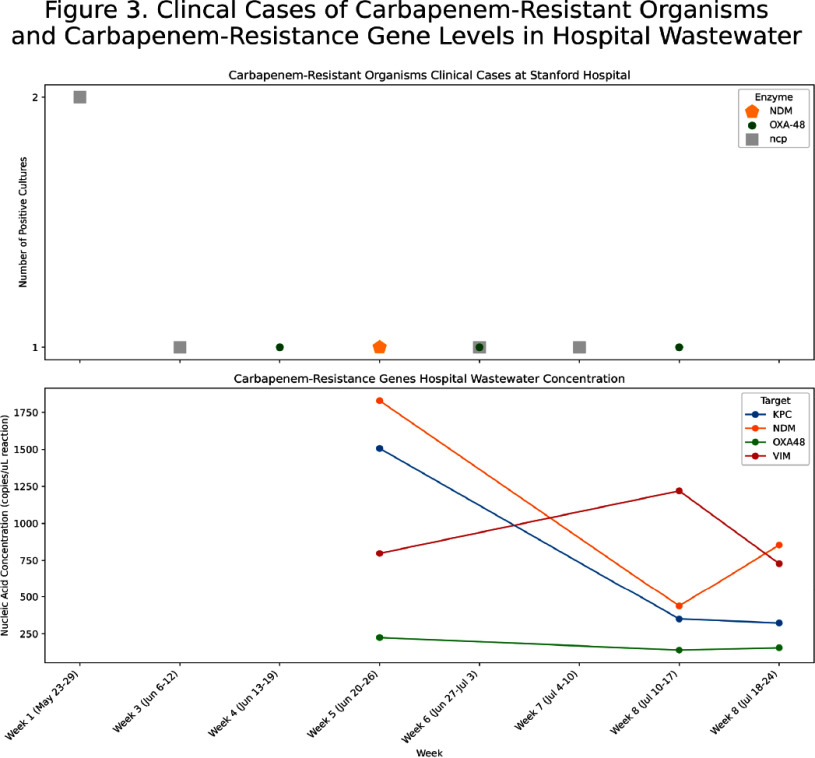

**Figure:**